# The Potential of Metatranscriptomics for Identifying Screening Targets for Bacterial Vaginosis

**DOI:** 10.1371/journal.pone.0076892

**Published:** 2013-09-27

**Authors:** Jimmy Twin, Catriona S. Bradshaw, Suzanne M. Garland, Christopher K. Fairley, Katherine Fethers, Sepehr N. Tabrizi

**Affiliations:** 1 Department of Microbiology and Infectious Diseases, The Royal Women's Hospital, Melbourne, Australia; 2 Murdoch Childrens Research Institute, Melbourne, Australia; 3 Melbourne Sexual Health Centre, Melbourne, Australia; 4 Department of Epidemiology and Preventive Medicine, Monash University, Melbourne, Australia; 5 Melbourne School of Population Health, University of Melbourne, Melbourne, Australia; 6 Department of Obstetrics and Gynaecology, University of Melbourne, Melbourne, Australia; 7 Department of Microbiology, The Royal Children's Hospital, Melbourne, Australia; Institute for Genome Sciences, University of Maryland School of Medicine, United States of America

## Abstract

**Background:**

The ribosomal RNA content of a sample collected from a woman with bacterial vaginosis (BV) was analysed to determine the active microbial community, and to identify potential targets for further screening.

**Methodology/Principal Findings:**

The sample from the BV patient underwent total RNA extraction, followed by physical subtraction of human rRNA and whole transcriptome amplification. The metatranscriptome was sequenced using Roche 454 titanium chemistry. The bioinformatics pipeline MG-RAST and desktop DNA analysis platforms were utilised to analyse results. Bacteria of the genus *Prevotella* (predominately *P. amnii*) constituted 36% of the 16S rRNA reads, followed by *Megasphaera* (19%), *Leptotrichia/Sneathia* (8%) and *Fusobacterium* (8%). Comparison of the abundances of several bacteria to quantitative PCR (qPCR) screening of extracted DNA revealed comparable relative abundances. This suggests a correlation between what was present and transcriptionally active in this sample: however distinct differences were seen when compared to the microbiome determined by 16S rRNA gene amplicon sequencing. To assess the presence of *P. amnii* in a larger pool of samples, 90 sexually active women were screened using qPCR. This bacterium was found to be strongly associated with BV (P<0.001, OR 23.3 (95%CI:2.9–190.7)) among the 90 women.

**Conclusions/Significance:**

This study highlighted the potential of metatranscriptomics as a tool for characterising metabolically active microbiota and identifying targets for further screening. *Prevotella amnii* was chosen as an example target, being the most metabolically active species present in the single patient with BV, and was found to be detected at a high concentration by qPCR in 31% of cohort with BV, with an association with both oral and penile-vaginal sex.

## Introduction

The advent of molecular-based screening utilising massively parallel DNA sequencing has increased our capability to characterise the microbial ecology of human clinical samples, both rapidly and economically [1]. In addition it has allowed a better understanding of the normal endogenous microbiota. The vaginal microbiota is complex, varying at different stages of reproductive life, as well as during the menstrual cycle. Bacterial vaginosis (BV) is a condition affecting the vaginal microbiota where in the childbearing age woman the natural microbiota (typically *Lactobacillus* spp.) is depleted, and replaced by an overgrowth of mixed, primarily anaerobic bacteria [2]. This condition has significant associations with miscarriage, premature birth and pelvic infections, and can increase a woman's risk of acquiring sexually transmitted infections and HIV [3]. No single aetiological agent has been identified yet for BV, and is now generally considered likely to be of polymicrobial aetiology.

BV is typically diagnosed using either the Nugent scoring method [4] that examines bacterial composition via a Gram smear or the Amsel criteria [5] that considers factors such as presence of discharge, amine production, presence of clue cells and a vaginal pH greater than 4.5. Through microbiome studies, the microbiology of BV has been better characterised. These studies have generally been carried out using PCR-derived 16S rRNA gene fragments, using “universal” bacterial 16S rRNA gene primers [2]. While this approach is able to provide a comprehensive understanding of the bacterial community membership, it is not able to determine which members are transcriptionally active.

Metatranscriptomics is the analysis of the RNA transcripts being expressed by a community at a given point of time. For bacteria, the predominant RNA classes are ribosomal (primarily 16S rRNA and 23S rRNA), which are able to be used as taxonomic identifiers. The metatranscriptomic screening of clinical samples has been described for gut and dental microbiomes [6], [7], with only one study to date applying this methodology to BV[8]. An important difficulty faced with the application of this technique to clinical samples is the low levels of RNA present in many samples and the overabundance of host RNA. This requires reduction of host RNA and subsequent linear amplification of RNA for transcriptomic analysis [9], [10].

This study describes a method to analyse microbial rRNA from vaginal samples that is applicable to variety of clinical sample types. It demonstrates how the data gained can provide valuable information for larger qPCR based screening studies.

## Materials and Methods

### Sample collection

A 26 year old woman presenting with abnormal vaginal discharge, odour, 4/4 Amsel criteria and a Nugent score of 10 was recruited with written consent from Melbourne Sexual Health Centre (MSHC), Victoria, Australia. This patient reported no recent antibiotic use, had no recent male sexual partner, and reported only having one female partner in the prior 3 months and three in the past 12 months. Vaginal discharge was collected using ten flocked swabs and immediately rotated and pooled in 4 ml RNAlater (Life Technologies, Grand Island, USA). The RNAlater solution was then stored at −80°C until processing.

For the second component of this study, extracted DNA (stored at −30°C) was utilised from a randomly selected subset of 90 samples obtained from a cross sectional study of sexually active women attending MSHC in 2003/4 [11]. This set of samples comprised 34 women with normal microbiota (Nugent 0–3), 20 with intermediate microbiota (Nugent 4–6) and 36 with BV (Nugent 7–10). Written consent for all de-identified participants and ethical approval for this study was obtained previously from The Alfred Hospital Human Research Ethics Committee.

### Nucleic acid preparation

**Figure 1:**
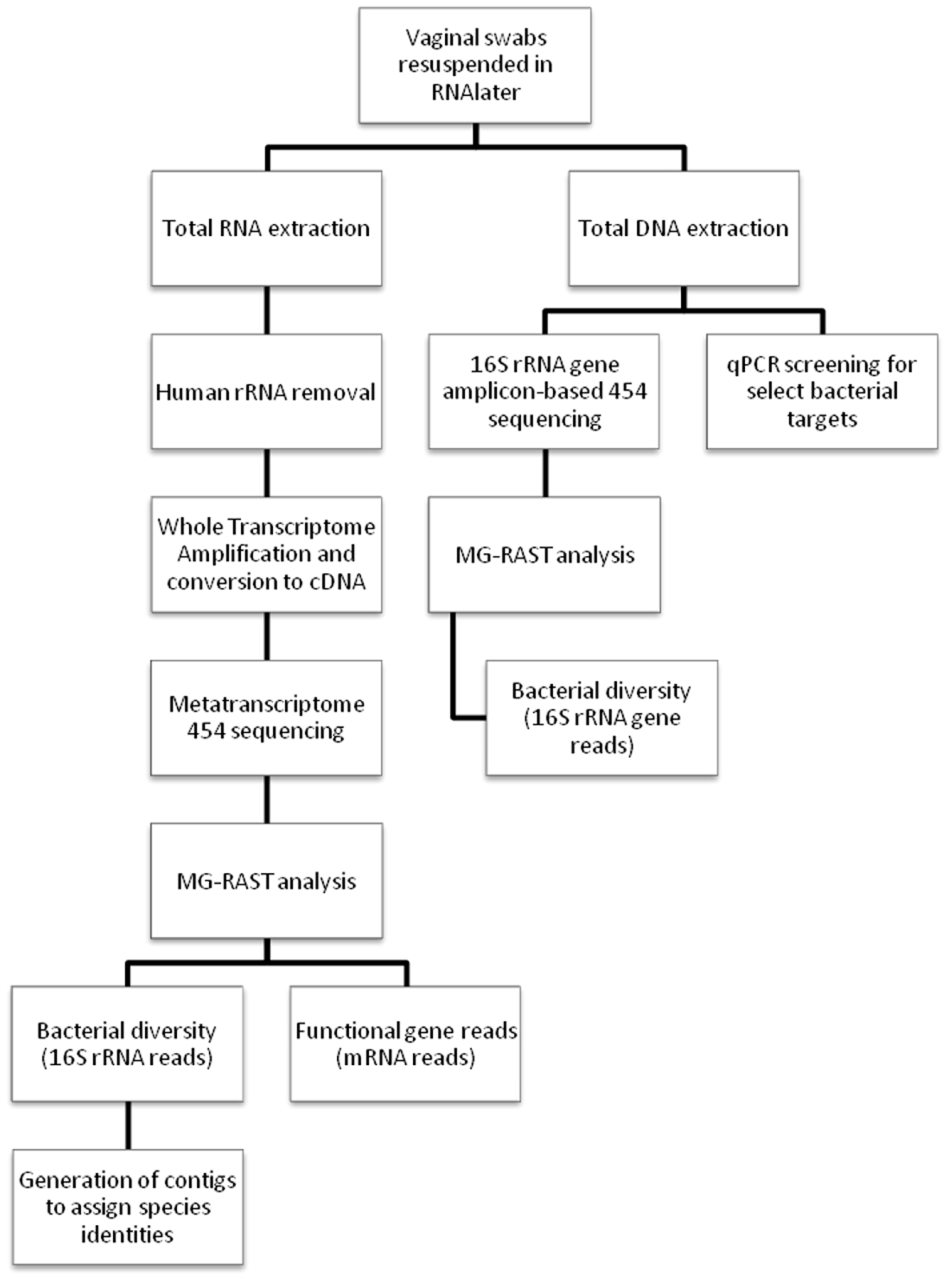
Overview of experiment. Flow chart of the work plan for this study.

Figure 1 provides an overview of the workflow for this study. Initially, total RNA was extracted from 2 ml RNAlater solution [12]. Total RNA extraction was performed using Trizol (Life Technologies) with use of 1-Bromo-3-chloropropane in place of chloroform followed by RNA purification using a RNeasy column (Qiagen, Hilden, Germany) [13]. Any remaining DNA was digested using the Turbo DNA-free enzyme (Life Technologies).

Human ribosomal content was subtracted using the MICROBEnrich Kit (Life Technologies) as per manufacturer instructions followed by removal of small RNAs with the MEGAclear kit (Life Technologies). RNA concentration was determined by Agilent 2100 Bioanalyzer (Agilent Technologies Inc., Santa Clara, USA). To increase the RNA content needed for sequencing, whole transcriptome amplification (WTA) and reverse-transcription using the TransPlex® Complete Whole Transcriptome Amplification Kit (Sigma-Aldrich Corporation, St. Louis, USA) was utilized. This resulted in a cDNA concentration of 50 ng/µl (from original RNA content of 3.5 ng/µl) with 2 µg submitted to AGRF (Australian Genome Research Facility Ltd., Brisbane, Australia) for 454 sequencing (FLX titanium chemistry; Roche/454 Life Sciences, Branford, USA).

Genomic DNA was precipitated from the remaining Trizol buffer using 0.3 ml of 100% ethanol per 1 ml of Trizol buffer followed by two washes of the DNA pellet with 0.1 M trisodium citrate in 10% ethanol [14]. An amplicon-based metagenomic library was generated from the extracted DNA using the universal bacterial PCR primers 27F and 338R that target the V1–V2 hypervariable regions of the 16S rRNA gene as previously described [15]. Both cDNA and 16S rRNA gene amplicon libraries were subsequently bar-coded using MID tags and sequenced together utilising a quarter region of a 454 sequencing plate.

### Sequence analysis

Subsequent to sequencing, all raw sequencing data was de-multiplexed according to their MID tags and the data obtained from each sample was then imported into Genomics Workbench version 4.5.1 (CLC Bio, Aarhus, Denmark), for removal of WTA primers and random primer sequences from each applicable read and data was filtered (low quality score limit of 0.05; maximum 2 ambiguous nucleotides allowed; minimum sequence length of 100 nt). Each dataset was then screened for chimeric sequences using UCHIME [16], with the trimmed and filtered data submitted to the MG-RAST (http://metagenomics.anl.gov) bioinformatics pipeline for analysis (cDNA library MG-RAST ID = 4461586.3; DNA amplicon MG-RAST ID = 4461792.3) [17]. A 90% cut-off was used for database searches within MG-RAST as an arbitrary cut-off for genus identities using the RDP database, and a 98% cut-off was used for species level identification [18]. MG-RAST generates abundance counts based on the number of unique hits a particular sequence has against a particular database. It is therefore highly likely that a single read may have multiple abundance counts assigned to it if there is an equal relatedness. The identities for each read were sorted using Excel 2007 (Microsoft Corporation, Redmond, USA) to eliminate multiple identical hits for individual reads, with manual analysis being carried out using the BLAST algorithm for discrepant samples. Graphical representation of bacterial abundances was achieved using Krona charts [19]. Functional genes from the cDNA library were characterised by SEED analysis within MG-RAST.

Reads derived from the cDNA library assigned to each major genus (comprising >10% of total population) using MG-RAST were also imported into Lasergene 8 (DNASTAR Inc., Madison, USA) for manual sequence alignment was carried out using a 98% sequence match cut-off. The consensus sequences of alignments were assigned an identity using the BLAST algorithm with a ≥98% identity required to assign a species name.

### Quantitative PCR (qPCR) screening

All assays were performed on the LC480 real-time instrument (Roche Diagnostics, Mannheim, Germany) using 5 µl DNA in a 20 µl reaction. The bacteria targeted were *Atopobium vaginae*, *Leptotrichia*/*Sneathia* spp., *Megasphaera* type I [20], *Gardnerella vaginalis* [21], and the genera *Prevotella* [22] and *Lactobacillus* [23]. A primer set for *Prevotella amnii* was developed using the forward and reverse primers 27F and 338R [24] with the incorporation of a TaqMan probe 5′-[6FAM] AAA GTT GGC CTA ATG CCC TAT G[BHQ1]-3′, specific to *P. amnii*, based on 454 sequencing data from this study and all available relevant sequences on the Genbank database. The total bacterial content of each sample was determined by the modification of an assay by Nikkari et al. (2002) [25] with the incorporation of a TaqMan probe, 516F (5′-[6FAM] TGC CAG CAG CCG CGG TAA [BHQ1]-3′) to calculate relative bacterial abundances and to determine sample adequacy for stored DNA samples.

### Statistical analyses

Relative bacterial abundances were compared using a paired *t* test or chi square test. The association of *P. amnii* with BV, defined as a Nugent score of 7–10, was made using a chi square test, reported with odds ratios (OR) with 95% confidence intervals (CI). All analyses were carried out using Stata version 12 (StataCorp LP, College Station, USA) [26].

## Results

### Nugent 10 patients' cDNA library

**Figure 2:**
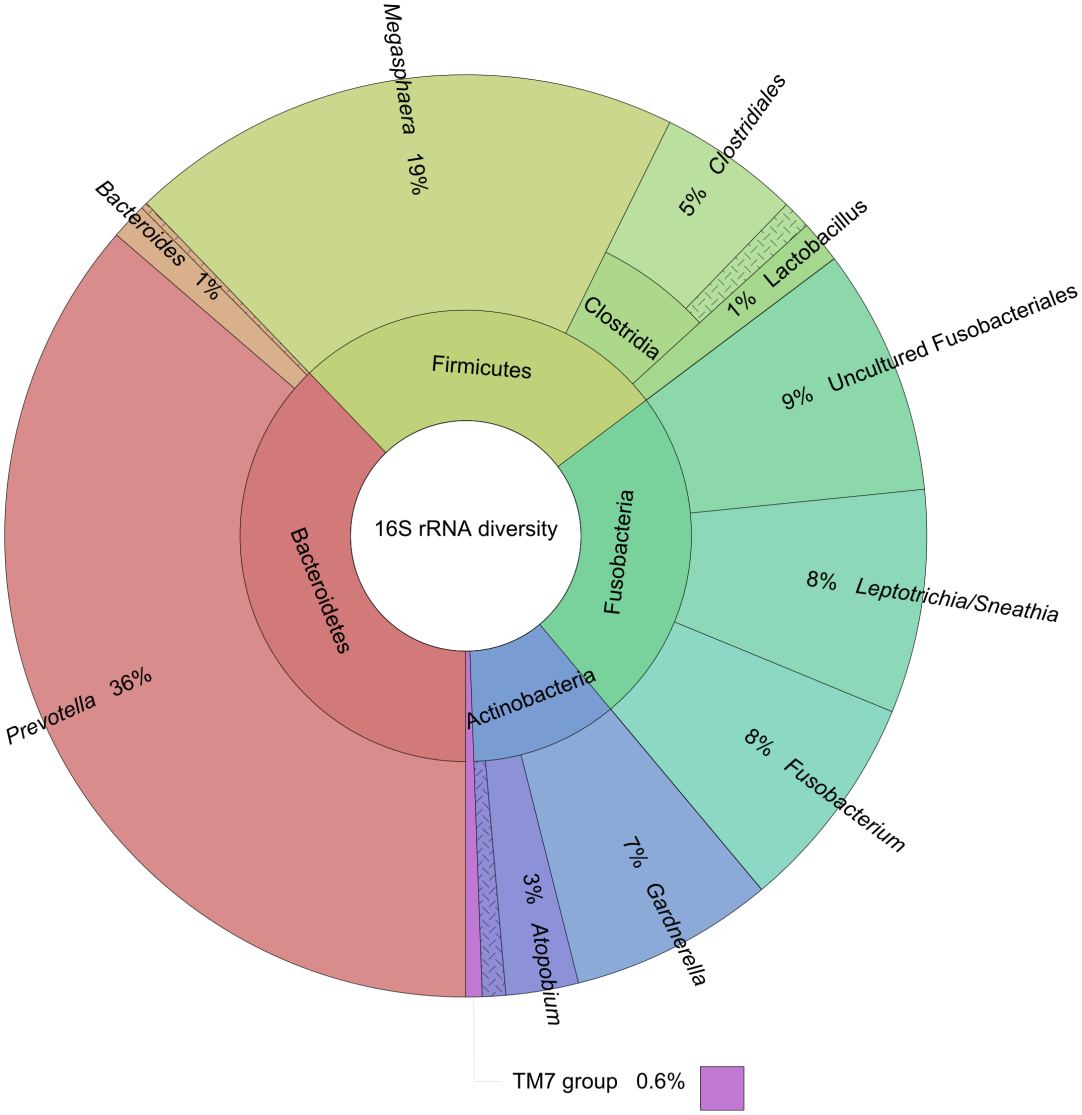
Bacterial diversity of cDNA library. Graphical representation of the bacterial genera identified through 16S rRNA gene matches for the Nugent = 10 cDNA using the RDP database search tool in MG-RAST.

In total, 89969 raw sequencing reads were obtained with an ultimate post data filtration of 78285 reads (average length  = 268±91 bp; GC content  = 49±5%). Overall, this library consisted of 72826 (93%) bacterial reads, 3865 (4.9%) human reads and 34 (0.04%) either plastid, fungal or viral reads (Table 1). Of the bacterial reads, 31857 (43.7%) were of the 16S rRNA gene and 4349 (6.0%) were non-ribosomal RNA (eg. mRNA) with the remaining matching other ribosomal regions such as the 23S rRNA gene and intergenic regions. Of the 16S rRNA gene reads, the most dominant genus identified was *Prevotella* (36% of the reads), followed by the genera *Megasphaera* (19%), *Leptotrichia*/*Sneathia* (8%), *Fusobacterium* (8%), and 9% of the reads matching uncultured members of the Fusobacteriales (Figure 2). Of the *Prevotella* reads, 78% formed contigs of ≥1000 bp in length that matched *P. amnii*. Analysis of assembly free reads of the *Prevotella* spp. reads at the species level (11725 reads with ≥98% match to the RDP database) showed similar results, however distinction between *P. amnii* and *P. bivia* was not possible for the majority of these sequences (data not shown). The major *Fusobacterium* was *F. nucleatum*, and the majority of *Megasphaera* reads were ≥98% similar to the currently uncharacterised *Veillonellaceae* bacterium S3PF24 (Genbank accession JX104009). The bioinformatics pipeline of MG-RAST was able to annotate 2053/4349 of the non-ribosomal RNA reads, which SEED analysis revealed to consist of genes involved primarily in protein metabolism (20.6%), carbohydrate/lipid utilisation (16.3%) and cluster based subsystems (13%). The most abundant functional gene read identities were primarily represented by genes expressed by the genus *Prevotella* (Table 2).

**Table 1:** Summary of Nugent 10 cDNA 454 sequencing reads analysed by MG-RAST.

Summary of MG-RAST reads	n	%
Bacterial	72826	93.0
16S rRNA gene	31857	40.7
Other ribosomal content*	36621	46.8
Functional genes	4348	5.6
Viral^†^	1	-
Fungal^‡^	4	-
Plastid	29	0.4
Human	3865	4.9
Poor quality^§^	1560	2.0
Total	78285	-

* 23S rRNA gene and intergenic regions; ^†^ human coronavirus; ^‡^ two reads of *Schizophyllum commune* cytochrome oxidase subunit 1 (*cox1*) gene, single reads of *Botryotinia fuckeliana* glycosyl hydrolase gene and Ascomycota hypothetical protein; ^§^ unable to be assigned to any taxonomy

**Table 2:** Top 20 Bacterial non-16S/23S gene Nugent 10 cDNA reads using 80% identity with a minimum alignment of 50 amino acid residues (n = 1361).

	All Bacteria		*Prevotella* spp.
Function	# reads	%	% of reads
TonB-dependent receptor plug domain protein	103	7.57	100
putative lipoprotein	76	5.58	100
glyceraldehyde-3-phosphate dehydrogenase, type I	57	4.19	87.7
MotA/TolQ/ExbB proton channel family protein	40	2.94	100
translation elongation factor Tu	39	2.87	46.2
phosphoenolpyruvate carboxykinase (ATP)	39	2.87	100
50S ribosomal protein L27	30	2.20	100
translation elongation factor G	32	2.35	59.4
TonB-dependent receptor	24	1.76	100
ExbD/TolR family protein	32	2.35	100
phosphoenolpyruvate carboxykinase	19	1.40	94.7
ribosomal protein L28	21	1.54	100
ribosomal protein S12	18	1.32	100
M16 family peptidase	16	1.18	100
D-phosphoglycerate dehydrogenase	15	1.10	100
glyceraldehyde 3-phosphate dehydrogenase	26	1.91	96.2
ribosomal protein L6	14	1.03	42.9
30S ribosomal protein S13	14	1.03	28.6
30S ribosomal protein S9	13	0.96	100
chaperone protein DnaK	13	0.96	0

### Comparison of cDNA to DNA from Nugent 10 patient

All of the predominant bacteria with >1% total abundance identified in the cDNA library were also detected using the 16S rRNA gene amplicon-based approach on extracted DNA. There was a significant difference in the relative abundances given between both libraries for the most dominant taxa, such as the genus *Lactobacillus* (primarily *L. iners*) that comprised 1.4% of the cDNA reads and 23% of the DNA amplicon library (p<0.0001). Also of particular note were reads pertaining to the genus *Gardnerella* that were present in 7% of the cDNA library, but were nearly non-existent in the DNA amplicon library with only seven reads detected (0.006%). When comparing the relative abundances of the seven taxa screened for using qPCR to the abundances given for the cDNA library, there was no significant difference (p = 0.9093). We found that the proportion of *Lactobacillus* spp. determined by qPCR to be 1.9% (cDNA = 1.4%; DNA amplicon = 23.1%), and the proportion of *Prevotella* spp. was 48% (cDNA = 35%; DNA amplicon = 11%) (Table 3).

**Table 3:** Comparison between Nugent 10 RNA and DNA bacterial abundances.

	RNA	DNA	
Taxa	cDNA library	16S rRNA (454)	qPCR
*Prevotella*	35.0%	11.0%	47.7%
*Megasphaera*	18.8%	8.7%	
*Uncultured Fusobacteriales*	8.3%	-	
*Leptotrichia/Sneathia*	7.5%	1.8%	6.1%
*Fusobacterium*	7.4%	2.0%	
*Gardnerella spp.*	6.9%	<1%	6.53%
*Clostridiales*	4.8%	1.3%	
*Atopobium spp.*	2.4%	19.0%	2.0%
*Lactobacillus spp.*	1.4%	23.1%	1.9%
*Bacteroides*	1.2%	2.6%	
*Mobiluncus*	0.8%	20.4%	
*TM7 group*	0.5%	5.4%	
*Eubacterium*	0.4%	-	
*Butyrivibrio/Uncultured Lachnospiraceae*	0.3%	2.8%	
*P. amnii*	*28.06%*		15.8%
*Megasphaera* Type I	*0.13%*		0.02%

### qPCR screening of sexually active women

*P. amnii* was detected in 11/36 (30.6%) women possessing a Nugent score of 7–10, whereby nine had a Nugent score of 7 and two with a Nugent score of 10. This bacterium was also detected in a single case possessing intermediate microbiota with a Nugent score of 6, and in none of 34 cases of normal microbiota (Nugent 0–3) (p<0.001; Table 4). The bacterial load of *P. amnii* in these 12 positive cases was high, averaging 1.41×10^9^ copies per swab (range = 7.74×10^6^ to 4.66×10^9^). This bacterium was found to be strongly associated with BV (*p*<0.001, OR 23.3 (95%CI:2.9–190.7)) among the 90 women. When the 90 women were stratified by different sexual exposures the association between BV and *P. amnii* persisted for different sexual exposures in men. While the associations between BV and *P. amnii* were not significant among women reporting receptive oral sex with a woman or sex with a woman in the last 3 months, these analyses involved only small numbers (Table 4).

**Table 4:** Prevalence of *Prevotella amnii* in cohort of sexually active women (n = 90).

	Nugent category					
	0–3	4–6	7–10	Total	*p* value*	OR (Nugent 7–10)
Male Sexual Partner (3 months)	0/25 (ND)	1/19 (5.3%)	11/30 (36.7%)	12/74 (16.2%)	<0.001	24.89 (95%CI:3.00–206.81)
Vaginal Sex (3 months)	0/24 (ND)	1/19 (5.3%)	11/30 (36.7%)	12/73 (16.4%)	<0.001	24.32 (95%CI:2.93–202.10)
Receptive Oral Sex (3 months)	0/25 (ND)	1/16 (6.3%)	11/31 (35.5%)	12/72 (16.7%)	0.001	22.00 (95%CI:2.65–182.61)
Receptive Male Oral Sex (3 months)	0/20 (ND)	1/15 (6.7%)	11/30 (36.7%)	12/65 (18.5%)	0.002	19.68 (95%CI:2.36–164.44)
Female Sexual Partner (3 months)	0/11 (ND)	0/2 (ND)	2/16 (12.5%)	2/29 (6.9%)	0.418	4.66 (95%CI:0.20–106.01)^†^
Receptive Female Oral	0/10 (ND)	0/2 (ND)	2/12 (16.7%)	2/24 (8.3%)	0.336	5.95 (95%CI:0.26–138.25)^†^
Total	0/34 (ND)	1/20 (5%)	11/36 (30.6%)	12/90 (13.3%)	<0.001	23.320 (95%CI:2.85–190.74)

* Chi square for proportion with *Prevotella amnii* in the three Nugent categories; †Haldane's estimation; OR =  Odds Ratio; ND = Not Detected.

## Discussion

This study explored the active microbial diversity of a vaginal sample taken from a lesbian woman with symptomatic BV to demonstrate the potential of metatranscriptomics for identifying targets for screening studies. *Prevotella amnii* was found to be the most active species present in this sample and was also detected at a high concentration by qPCR in 30.6% of BV cases in sexually active women. Although we identified *P. amnii* as the predominant *Prevotella* species in this sample (78% of *Prevotella* reads; 28.1% of total bacterial population), there may be a possible overestimation in the cDNA abundance given, as conserved regions shared by multiple *Prevotella* spp. may be involved through chimeric assembly. This must be kept in mind when comparing to qPCR data (33.1% of *Prevotella* reads; 15.8% of total bacterial population) that would otherwise suggest that this species is more active than others in this sample. This bacteria has only recently been associated with BV in the literature [27]. Its closest relative, *P. bivia*, is highly cited as being associated with this condition, and *in vitro* studies have shown this bacterium to form a symbiotic relationship with *G. vaginali*s [28]. These two *Prevotella* spp. share many phenotypic traits and it has been noted that culture-based methodologies may not differentiate between these two species, with full length 16S rRNA gene sequencing recommended for species differentiation [29].

The other predominant bacteria in this case were identified through 16S rRNA gene as the currently uncharacterised isolate *Veillonellaceae* bacterium S3PF24 (phylogenetically belonging to the genus *Megasphaera*) that was originally isolated from a vaginal environment, and the bacterium *Fusobacterium nucleatum*. *F. nucleatum* has been described in cases of BV previously [30]. Interestingly it has been implicated in amniotic infection and pre-term birth in cases where the bacterium was also detected in the patients' oral cavity [31], [32]. *F. nucleatum*, as well as both *Prevotella* and *Megasphaera* spp. are regularly associated with periodontal disease [33], [34], [35], and it is of interest to note that the single Nugent score 10 participant analysed in this study had a history of recent female-female oral sex. These three bacteria were more abundant than the usual predominant *G. vaginalis* and *A. vaginae* in the cDNA library, which collectively only comprised 10% [2]. Further work is warranted to determine the role of these bacteria, in particular *P. amnii*, in the pathogenesis of BV. Of particular interest is whether their association with oral sex represents a possible route of transmission.

Although not a focus of this study, mRNA reads were also identified in this study that were primarily associated with protein metabolism and carbohydrate/lipid utilisation pathways of the genus *Prevotella*. The low abundance of mRNA reads (5.6%) was not surprising, given the low general relative abundance of mRNA in relation to total RNA in bacterial cells [36]. To focus more upon this class of RNA transcripts, an additional enrichment step, such as the removal of bacterial rRNA can be employed [37], in the same manner as human rRNA was reduced in this study.

A comparison was made between the cDNA and DNA amplicon libraries to determine what proportion of the microbial community was active, and differences were observed between these libraries. This may suggest that there was a significant difference between what is present and what is transcriptionally active, although these findings are limited by the fact that this is a single sampling point. The broad range amplification of bacterial DNA using the 16S rRNA gene may have potential bias in the community structure, as has been found in numerous studies [38], [39], [40], [41]. Our data suggests that *G. vaginalis* was underrepresented in the DNA amplicon library when compared to the qPCR data from the same DNA sample. Despite being one of the most utilized primers for microbiome analysis, there has been suggestion in the literature that the forward PCR primer 27F may not reliably amplify this species [42], and our analysis has demonstrated this similarly through qPCR for *G. vaginalis*. Also, given that *L. iners* was found to be underrepresented in the cDNA library when compared to the DNA amplicon library, we applied a genus-specific *Lactobacillus* qPCR assay to the DNA sample, as well as one that targeted the genus *Prevotella* that was present at a much higher abundance in the cDNA library. We found that there was no statistical difference in the abundances given between qPCR and the cDNA library for these genera. Based on this limited data, we can suggest misrepresentation of several taxa in the DNA amplicon library occurred, and that in this case, the vast majority of the bacteria physically present in this sample were also transcriptionally active. This further highlights the advantage of transcriptomics for such analysis as our data suggest that this analysis gives better representation of bacterial levels across the board.

MG-RAST for the analysis of metatranscriptomic work can be carried out using a standard desktop or laptop computer. However, this method may lead to misclassification of certain reads. For example, in this study it was found that many *Gardnerella* reads were being classified as the genus *Bifidobacterium*, *Megasphaera* reads as the genera *Veillonella* and *Dialister*, and those of the genera *Leptrotrichia*/*Sneathia* as *Streptobacillus*. Therefore care must be taken in the interpretation of results, and manual checking of representative reads is required to ensure an accurate identification of sequencing reads to the genus level.

In summary, this study has shown how metatranscriptomics can be utilised as a useful tool to carry out an in-depth examination of the active microbial ecology of an environment, and is capable of generating more taxonomic and potential functional data than an amplicon sequencing-based approach alone, and avoid potential misrepresentation issues caused by PCR primer selection. We have demonstrated that this kind of metatranscriptomic approach can be used to identify targets for screening in larger studies. As this study focussed on a single individual, generalisations based on the data are not possible and transcriptomic analyses on a large cohort is warranted. This being said, our limited findings reinforce that *P. amnii* may be an important BV-associated bacterium, and should be included in future studies investigating the pathogenesis of BV. Its previously known association with periodontal disease, and the association with oral sex in this study, make it interesting to speculate about transmission of pathogenic bacteria between the oral and vaginal cavities, and their role in the development of BV.
